# A Disgraceful Act

**Published:** 1886-02

**Authors:** 


A DISGRACEFUL ACT.—*The Philadelphia Medical Bulletin* says the Philadelphia Medical Society consists of some five hundred members; that in October last, in accordance with the provisions of the by-laws, a list of delegates to the American Medical Association had been nominated, and that recently a minority of the Society got together, set aside the regular ticket, and substituted one known to be in the interest of the "bolters," thus riding over and trampling under foot the Constitution and By-Laws of the Society; and disregarding all precedent, elected these men, some of whom had been, for months, suspended for nonpayment of dues, and others were not members at all. The scene is described as having been boisterous, and was characterized by the cat-calls, and other concomitants of a political ward meeting. For shame! We look to see the majority assert its rights, and no doubt right will prevail in the end.

